# A Review and Characterization of the Various Perceptions of Quality Cancer Care

**DOI:** 10.1002/cncr.25644

**Published:** 2010-10-11

**Authors:** Ann D Colosia, Gerson Peltz, Gerhardt Pohl, Esther Liu, Kati Copley-Merriman, Shahnaz Khan, James A Kaye

**Affiliations:** 1 Market Access and Outcomes Strategy, RTI Health SolutionsResearch Triangle Park, North Carolina; 2 Global Health Outcomes Oncology, Eli Lilly and CompanyIndianapolis, Indiana; 3 Market Access and Outcomes Strategy, RTI Health SolutionsAnn Arbor, Michigan; 4 Epidemiology, RTI Health SolutionsWaltham, Massachusetts

**Keywords:** quality of healthcare, quality indicators, quality assurance, neoplasms/drug therapy, cancer care facilities, hospital oncology service

## Abstract

**BACKGROUND:**

It is important to maintain high-quality cancer care while reducing spending. This requires an understanding of how stakeholders define “quality.” The objective of this literature review was to understand the perceptions patients, physicians, and managed care professionals have about quality cancer care, especially chemotherapy.

**METHODS:**

A computerized literature search was conducted for articles concerning quality cancer care in patients who received chemotherapy. Among >1100 identified sources, 25 presented interviews/survey results from stakeholders.

**RESULTS:**

Patients defined quality cancer care as being treated well by providers, having multiple treatment options, and being part of the decision-making process. Waiting to see providers, having problems with referrals, going to different locations for treatment, experiencing billing inaccuracies, and navigating managed care reimbursement negatively affected patients' quality-of-care perceptions. Providers perceived quality cancer care as making decisions based on the risks-benefits of specific chemotherapy regimens and patients' health status rather than costs. Providers objected to spending substantial time interacting with payers instead of delivering care to patients. Payers must control the costs of cancer care but do not want an adversarial relationship with providers and patients. Payers' methods of managing cancer more efficiently involved working with providers to develop assessment and decision-assist tools.

**CONCLUSIONS:**

Delivering quality cancer care is increasingly difficult because of the shortage of oncologists and rising costs of chemotherapy agents, radiation therapy, and imaging tests. The definition of quality cancer care differed among stakeholders, and healthcare reform must reflect these various needs to maintain and improve quality while controlling costs. Cancer 2011. © 2010 American Cancer Society

Leading cancer experts and organizations in the United States are seeking ways to assess quality cancer care and ensure its delivery because of increasing costs to patients and healthcare plans,^1^,^2^ including rising out-of-pocket costs for cancer medications.^3^ The anticipated shortage of oncologists^4^,^5^ also will challenge the delivery of quality cancer care. According to the Institute of Medicine, quality care should be consistent with current professional knowledge about a disease, including its diagnosis, staging, and treatment, and should produce the desired health outcomes.^6^ The objective of this literature review was to identify the perceptions of stakeholders—providers, patients, and payers—of quality cancer care in the United States, particularly for patients who receive chemotherapy. On the basis of these perceptions, this review offers several recommendations for improving the quality of cancer care.

## MATERIALS AND METHODS

**Figure 1:**
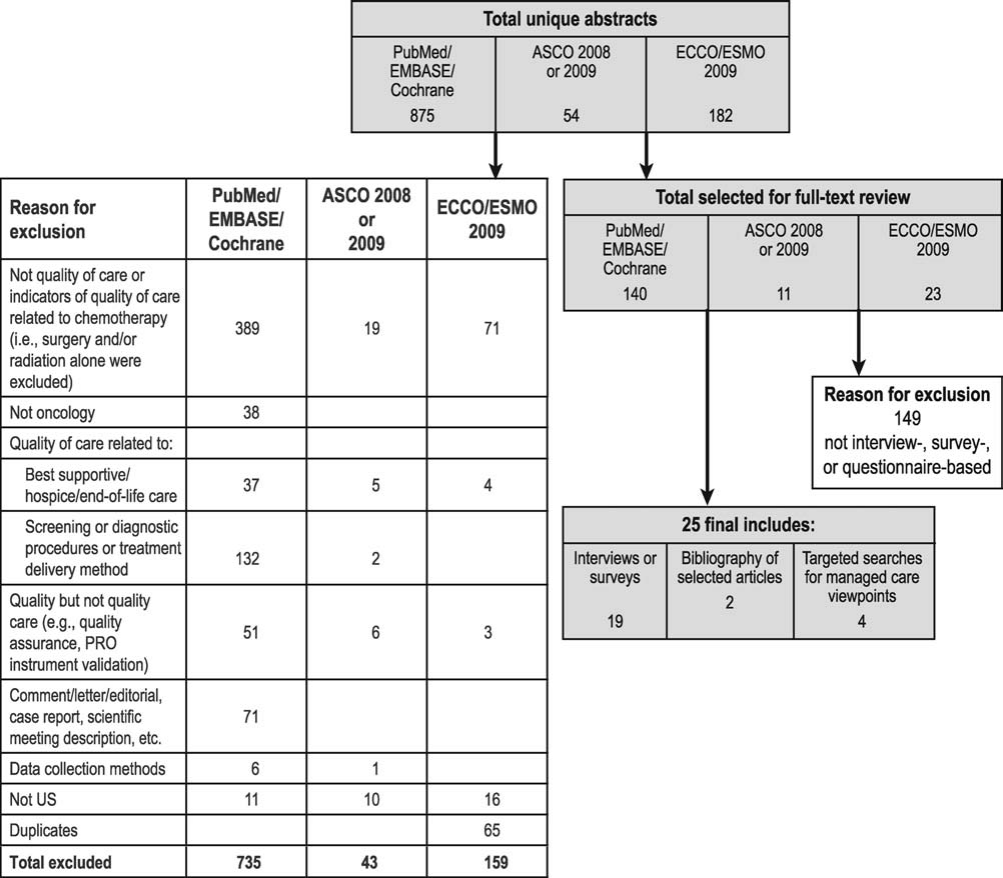
This flow diagram shows the number of abstracts and full-text sources reviewed for the current literature review and reasons for exclusion. EMBASE indicates Excerpta Medica Database; ASCO, American Society of Clinical Oncology; ECCO, European Cancer Organization; ESMO, European Society for Medical Oncology; PRO, patient-reported outcome.

The PubMed database, the Excerpta Medica Database (EMBASE), and Cochrane Reviews were searched systematically for publications related to cancer. PubMed medical subject heading (MeSH) terms included “Neoplasms/Drug Therapy”; “Neoplasms/Radiotherapy”; “Neoplasms/Surgery”; “Cancer Care Facilities”; “Oncology Service, Hospital”; and “Radiation Oncology.” MeSH terms to capture studies on quality of care were “Quality of Health Care”; “Quality Assurance, Health Care”; and “Quality Indicators, Health Care.” For this study, we reviewed 875 article abstracts and applied inclusion/exclusion criteria (Fig. 1). The primary focus of the study was quality of cancer care for patients who were receiving chemotherapy. Of the 875 article abstracts, 140 publications of interest were obtained for full review. Two hundred thirty-six conference abstracts also were identified from the American Society of Clinical Oncology 2008 and 2009 meetings and from the European Cancer Organization/European Society of Medical Oncology 2009 meeting. The conference abstracts were searched using the following terms: “quality of healthcare,” “quality of healthcare,” “quality assurance,” “quality indicators,” “quality care,” and “quality of care.” Additional articles were obtained from the selected article bibliographies and from online searches informed by content from the selected articles. Online searches were needed in particular to obtain perspectives of managed care professionals. The Internet was searched for “managed care” with “journal” to identify publications that specifically addressed managed care, and then the tables of contents were searched when available online. To ensure a varied managed care perspective, this review included publications from professionals in the field that related their experiences and/or described their insurance companies' programs to manage cancer patients.

## RESULTS

Although the quality of cancer care was discussed frequently in conferences and publications, there were few structured studies that obtained patients' views, and even fewer reported interviews with providers and managed care professionals. In total, 25 sources described surveys or interviews with patients, providers, or professionals in managed care settings.

### Patient Perspectives

Thirteen studies reported patient perceptions of quality cancer care from >9000 patients (Table 1). Five of those 13 studies enrolled exclusively patients with breast cancer (N = 1039), whereas 1 study enrolled only patients with colorectal cancer (N = 1067). Four studies included 6905 patients with any cancer or with 1 of several kinds of cancer.

**Table 1:** Sources Reporting Patients' Perceptions of Quality Cancer Care

Study	Study Design	Type of Cancer in Patient Population	Sample Size
Liang 2002^7^	Interview	Breast	613
Goodwin 2003^8^	Interview	Breast	169
Brearley 2009^11^	Interview	Gastrointestinal	19
Bourjolly 2004^18^	Interview^a^	Breast	33
Wenzel & Steeves 2008^17^	Interview	Breast	14
Lunik 1996^12^	Interview	Lymphoma	1
Arora 2009^13^	Patient's recounting	Lymphoma	1
Gesell & Gregory 2004^15^	Survey/questionnaire	Any (16 types)	5907
Ayanian 2005^10^	Survey/questionnaire	Colorectal	1067
Franco 2009^42^	Survey/questionnaire	Breast	210
Williams 1998^9^	Instrument (HCI)	Any	259 (165 With cancer)
Nguyen 2009^43^	Instrument (EORTC QLQ-C30/ questionnaire)	Breast, prostate, lung, head and neck, rectal	686
Anderson & Zwelling 1996^16^	Instrument (SERVQUAL)	Breast, leukemia, gastrointestinal, any requiring bone marrow aspiration	147
Total no. of patients		9126 (9032 With cancer)	

HCI indicates Holistic Caring Inventory; EORTC QLQ-C30, European Organization for Research and Treatment of Cancer Quality-of-Life Questionnaire; SERVQUAL, service quality questionnaire.

a Bourjolly and colleagues also interviewed providers.

#### Promoters of and barriers to quality care: Information and interactions

Studies of patient perceptions revealed that patients report receiving high-quality cancer care when they are able to obtain information about their health and treatment, participate in decision making, trust their physician, communicate their feelings to providers, and believe that care from various professionals is well coordinated (Table 2). In a study among 613 women aged ≥67 years with breast cancer, surgeon-initiated communication and being presented with multiple treatment options gave patients the perception of having a choice of treatment.^7^ Women who reported high levels of surgeon-initiated communication were 2.13 times more likely to be satisfied with their care than women who reported low levels of surgeon-initiated communication. Provision of information from nurse case managers also led to a higher level of patient comfort. In a randomized, prospective trial of women aged ≥65 years with breast cancer, 169 women who were assigned a case manager were more likely to perceive that they had a real choice in their treatment compared with 166 women who did not have a case manager.^8^

**Table 2:** Patients' Perception of Relevant Factors for Quality Cancer Care

Factor	Reference Source
**Promoters of quality care**	
**Communication**	
Surgeon-initiated communication	Liang 2002^7^
Better patient-physician communication	Franco 2009^42^
Knowledge of which provider to approach with questions	Franco 2009^42^
Other (nonsurgeon) healthcare professionals as source of communication (eg, case manager)	Goodwin 2003^8^
Trust in physician (consistent across races)	Franco 2009^42^; Schrag 2005^44^
Caring behavior and attitudes from nurses (treated with care, gentleness, respect, and attention)	Williams 1998^9^
Better treatment by staff/staff courtesy/staff concern for patient comfort	Gesell & Gregory 2004^15^; Franco 2009.^42^
**Support**	
Strong support system of family and friends	Lunik 1996^12^; Arora 2009^13^; Weingart 2007^14^; Ashing 2003^45^
Spiritual support	Ashing 2003^45^
Multiple treatment options	Liang 2002^7^
Clean facility	Gesell & Gregory 2004^15^
**Barriers to quality care**	
Difficulty of attaining the cancer diagnosis	Lunik 1996^12^; Arora 2009^13^
Problems obtaining health information	Ayanian 2005^10^
Problems obtaining treatment information	Ayanian 2005^10^
Problems with psychosocial care/patient's emotional needs not addressed	Ayanian 2005^10^; Gesell & Gregory 2004^15^
Lack of involvement in decision making	Ayanian 2005^10^
Lack of coordination of care among providers	Ayanian 2005^10^
Lack of awareness, trust, and familiarity with healthcare system and lack of insurance or transportation among Asian-American women	Ashing 2003^45^
Unsafe care (adverse events, close calls, and medical errors)	Weingart 2007^14^
Service quality incidents (waits and delays in service, poor care coordination, communication issues resulting in lack of access to care)	Weingart 2007^14^; Anderson & Zwelling 1996^16^; Ayanian 2005^10^; Gesell & Gregory 2004^15^
Difficulty reaching staff	Gesell & Gregory 2004^15^
Billing inaccuracies	Anderson and Zwelling^16^
Difficulty with managed care tasks	Wenzel and Steeves^17^
Managing or mediating between the managed care organization and the cancer	Wenzel and Steeves^17^

Patients were concerned about their personal interactions with providers and were more likely to notice a lack of positive interactions than the presence of quality interactions.^9^ In an analysis of 3 studies and that included a total of 259 patients, mean responses to the Holistic Caring Inventory, which consists of physical, psychological, sociocultural, and spiritual domains, indicated that patients perceived that they received more physical and sensitive caring than interpretive and spiritual caring from nurses. Interpretive caring, as defined in the studies, required that the nurse have more conversations with the patient's family, share feelings and information, and be aware of specific circumstances of the patient's life to explain how cancer and treatment would affect these areas. Nurses may not have time to provide the depth of interactions required to offer spiritual care. Patients expressed that they did not expect spiritual care but did expect to be treated as a unique individual and with care, gentleness, respect, and attention.^9^

When information was difficult to obtain, trust was limited, or care was not well coordinated, patients experienced reduced quality of care. In a survey of >1000 patients with colorectal cancer, results across all racial and ethnic groups indicated that the domains of care with the highest problem scores (indicating poor perceptions) were reported for health information, treatment information, psychosocial care, and coordination of care. Lower problem scores (indicating better perceptions) were reported for confidence in providers and access to cancer care. Approximately 50% of patients reported receiving insufficient health information about changes in their work or usual activities, relationship with their spouse or partner, or sexual activity. Approximately 25% to 29% of patients reported problems that included lack of sufficient information regarding treatments, lack of coordination among providers, and lack of involvement in decision making. Among patients who were eligible for chemotherapy after surgery, those who did not receive it were more likely to report problems with confidence in their providers and access to care.^10^

Patient perceptions may change over time and with stage of disease. In a recent study in the United Kingdom, interviews with 19 patients who had gastrointestinal cancers revealed that, during the initial, acute treatment phases (diagnosis and 3 months after diagnosis), quality of life, daily function, and treatment-related symptoms were the most important issues for patients among those investigated. During the later phases (6 months and 12 months after diagnosis), patients' concerns shifted from worries about physical symptoms to worries about recurrence, lack of new treatment options, gaining independence, getting clear information on long-term care options, and social and financial survivorship.^11^

#### Support systems

An essential part of receiving quality cancer care for patients was feeling that they had a strong support system, consisting of family and friends who would ask difficult questions when the patients, for any reason, could not.^12^,^13^ In addition, family and friends helped patients avoid potential harm from medication errors, such as pointing out the existence of a drug allergy that was not noted in the patient records.^14^

#### Service quality

A survey of almost 6000 cancer outpatients who received treatment at 23 US hospitals assessed patients' experiences encompassed under the umbrella of service quality. The service quality areas with the highest scores (indicating patient satisfaction) were staff courtesy, cleanliness of the facility, and staff concern for patient comfort. The “likelihood of recommending the hospital's services to others,” which captured overall satisfaction with service quality, also received 1 of the highest scores. The areas with the lowest scores (indicating dissatisfaction) were waiting time, ease of reaching the office staff, and the degree to which the staff addressed the patient's emotional needs.^15^

Results from another service quality study indicated that, from a patient perspective, billing accuracy and waiting times were significant problems.^16^ The SERVQUAL questionnaire was administered to 200 patients at each of 4 clinics (N = 800): medical breast, leukemia, medical gastrointestinal, and bone marrow aspiration. Across all 4 clinics, the attribute of reliability (which included service provided at the time promised, sympathetic and reassuring manners, and billing accuracy) was rated consistently by patients as the most important attribute and also received the lowest ratings in terms of quality.^16^

#### Patient perceptions of US managed care quality of care

Interviews of patients with breast cancer revealed that interactions with managed care organizations (MCOs), including issues with billing accuracy, copayments, and referrals, were a major burden and affected perceptions of the quality of care.^17^ Two major themes emerged from this research. The first was that patients had “difficulty completing managed care tasks” (involving issues such as getting a referral; clarifying copayment levels; finding knowledgeable and helpful MCO staff; and getting assistance with paperwork, billing, and treatment approvals). In addition, patients perceived a lack of logical and caring decision making on the part of the MCO. Delays in determining out-of-pocket expenses resulted in distress and difficulty in planning future treatments. In addition to the physical vulnerability associated with their condition, patients were subject to a financial vulnerability related to payment for care, because they worried about discontinuation of coverage and inability to get further treatment if coverage was discontinued. Most patients expressed a lack of confidence that there would be improvements in the MCO system over time. The second major theme was problems in “managing or mediating between the MCO and the cancer [care providers],” in which patients perceived MCO management and cancer care as separate entities that had to be linked by the patient to achieve the best outcomes. Whereas some patients accepted assistance from the cancer center about billing issues when possible, others used a more take-charge approach and expressed frustration at the extra level of complication caused by this paperwork. In addition, patients felt burdened with having to coordinate between the provider and the payer while also taking care of their physical needs related to the disease.^17^

A study by Bourjolly and colleagues^18^ that involved interviews with 33 women who had breast cancer revealed 2 main areas of difficulty with insurance: obtaining referrals and receiving medical services in different locations. The patients' primary concern was finding the best care to treat their disease regardless of the type of insurance they had. Having to get referrals was regarded as a very frustrating experience. None of the women reported being denied any treatment options because of insurance restrictions; however, 1 woman's insurance refused to pay for a bone scan because of problems with the referral. Having various aspects of one's treatment in different locations based on insurance requirements was observed as an inconvenience. Although the women received chemotherapy or radiation treatment at a cancer center, some had to get mammograms and laboratory work at other locations.^18^

#### Patient characteristics associated with unfavorable perceptions of care quality

Three studies identified patient and clinical characteristics that were associated with unfavorable perceptions of quality care. These characteristics included factors related to health status, type of treatment, living conditions, and sociodemographic characteristics of the patients, including age, income level, ethnicity, and language spoken (Table 3).

**Table 3:** Predictors of Patient Dissatisfaction With Cancer Care

Predictive Factor	Instrument	Reference Source
Poor global health in patients with head-and-neck cancer	EORTC QLQ-C30 for sociodemographic, clinical characteristics, and quality of life data; OUT-PATSAT35 for satisfaction with providers, care organization, and services	Nguyen 2009^43^
Receipt of radiotherapy in patients with head-and-neck cancer		
Living alone^a^		
Young age (<55 y vs >55 y; for physicians' availability)		
High income (for provision of information from nurses)		
African-American race (associated with less trust in physician, perception of greater difficulty with psychosocial care, care coordination, access to care, obtaining health information)	Five-item questionnaire assessing physician communication (Franco 2009^42^); adapted Picker Institute survey (Ayanian 2005^10^)	Ayanian 2005^10^; Franco 2009^42^
Asian or Pacific Islander (associated with perception of greater difficulty with care coordination, access to care, obtaining health information)	Adapted Picker Institute survey	Ayanian 2005^10^
Nonwhite^b^		
Non–English-speaking^b^		

EORTC QLQ indicates European Organization for Research and Treatment of Cancer Quality of Life Questionnaire; OUT-PATSAT35, The EORTC Cancer Outpatient Satisfaction With Care Questionnaire.

a For provision of information from physicians and nurses.

b Less likely to rate overall quality of cancer care as excellent or very good.

### Provider Perspectives

Seven studies captured perspectives on aspects of quality cancer care from >2200 providers (Table 4), including 1 study that also surveyed patients.^18^

**Table 4:** Sources That Reported Providers' Perceptions of Quality Cancer Care

Reference	Study Design	Type of Provider	Type of Cancer in Patients Treated	Sample Size
Bickell 2007^22^	Interview	Surgeons	Breast	35
Bourjolly 2004^18^	Interview^a^	Physicians, nurses business office staff	Breast	10
Keating 2008^23^	Survey/questionnaire	Medical oncologists, surgeons	Colorectal	1096
Lamkin 2002^24^	Survey/questionnaire	Registered nurses specializing in oncology	Any	463
Tisnado 2008^26^	Survey/questionnaire	Medical oncologists, radiation oncologists, surgeons	Any	348
Katz 2009^19^	Survey/questionnaire	Surgeons	Breast	312
Genentech 2008^25^	Survey/questionnaire	Oncologists	Any	NR^b^
Total no. of providers				≥2264^b^

NR indicates not reported.

a Bourjolly and colleagues also interviewed patients.

b The survey for Genentech was mailed to 5000 medical oncologists; the response rate was low, but the number of responses was not reported.

#### Physician reports on quality indicators

Three studies were identified that asked physicians about multidisciplinary care, appropriate receipt of chemotherapy, and practice processes. A survey of 312 surgeons who treated breast cancer in 2 large metropolitan cities (Los Angeles and Detroit) revealed low use of patient and practice management processes. Approximately 10% of surgeons reported that ≥50% of their patients had multidisciplinary physician communication. Only 5% of surgeons reported that ≥50% of their patients were provided with presurgical decision and care support services (eg, viewing a video about breast cancer). Surgeons who specialized and surgeons in teaching programs were more likely to use practice management processes.^19^

Interviews were conducted with surgeons who treated women for primary stage I or II breast cancer to determine why 21% of the women in an earlier study^20^ did not receive adjuvant therapy that would have been consistent with then current National Comprehensive Cancer Network (NCCN) guidelines.^21^ Underuse of adjuvant therapy was highest among African-American women (34%), followed by Hispanic women (23%), and white women (16%). Of the original study's 37 surgeons, 35 were available to be interviewed about the underuse of adjuvant therapy for the 119 women they had treated (Table 5). For 34% of the women, surgeons did not recommend adjuvant therapy based on valid health considerations. Lack of physician knowledge was the underlying reason for adjuvant therapy underuse in 3% of patients.^22^ Another 31% of the women declined adjuvant therapy. The surgeons were asked about which practical barriers could have played a role in each of their patients' decisions to decline therapy, but the respondents often did not know whether patients had difficulty with various forms of support. For the 119 women who underused adjuvant chemotherapy, physicians did not know their circumstances in terms of financial support (45%), emotional support (58%), and social support (57%). The surgeons also often did not know whether patients were resistant to receiving adjuvant treatment (56%), understood the risks and benefits of adjuvant therapy (54%), or were unable to tolerate adjuvant therapy (52%).^22^

**Table 5:** Physician Responses to Questions About Chemotherapy Use

Study	Population Queried	Quality Measure/Outcome
Bickell 2007^22^	Surgeons (n=35) who treated 119 women for primary stage I or II breast cancer and who did not receive NCCN guideline-recommended adjuvant therapy	Reasons offered for not receiving guideline-recommended therapy: therapy was recommended but patients declined (31%); therapy was recommended but physician could not identify a reason for its not being administered (system failure; 34%); therapy was not recommended (all valid health reasons; 34%)
Keating 2008^23^	Physicians (n=1096) were queried about chemotherapy use in 6 case scenarios	Chemotherapy recommended

Keating 2008^23^		
Variable	Comorbidity	Chemotherapy Recommended, Adjusted % of Physicians
**Patient age, y**^a^		
55	None	99
80	None	92.6
55	Moderate CHF	88.6
80	Moderate CHF	47.3
55	Severe CHF	24.9
80	Severe CHF	8.9
*P*		<.001
**Physician age, y**^b^		
<40		62.6
40-49		62.2
50-54		62.1
55-59		58.3
≥60		54
*P*		<.001

NCCN indicates National Comprehensive Cancer Network; CHF, congestive heart failure.

a The strongest predictors of recommending chemotherapy were patient age and comorbidity.

b The strongest physician predictor for recommending chemotherapy was young age.

For the remaining 34% of patients, surgeons could not identify why adjuvant therapy was not administered. Among these patients, referral to an oncology clinic rather than a specific oncologist was the only risk factor identified for not receiving guideline-appropriate adjuvant therapy (odds ratio, 4.8; 95% confidence interval, 1.1-21.3; *P* < .01). Few of the surgeons had mechanisms in place to determine whether a patient followed through with a recommended referral or treatment. This may be because the US healthcare system is relatively nonpaternalistic and regards patients as autonomous decision makers who have the skills to determine their own care.^22^

In a physician survey that was conducted by Cancer Care Outcomes Research and Surveillance Consortium investigators, both patient and clinician characteristics influenced the decision to use chemotherapy for older or sicker patients (Table 5). In the study, 1096 physicians were asked whether they would recommend chemotherapy in 6 case scenarios of patients who underwent curative resection for stage III colon cancer. In linear regression analyses adjusting for physician and patient characteristics, the strongest predictors of chemotherapy recommendation were patient age (aged 55 years vs 80 years) and the level of comorbidity (none, moderate congestive heart failure, or severe congestive heart failure). Physicians were less likely to recommend chemotherapy when the patient was both sicker and older (*P* < .001) (Table 5). In adjusted analyses, younger physicians were more likely to recommend chemotherapy than older physicians.^23^

#### Workload and quality care

A survey in 2000 of 463 registered nurses (RNs) who specialized in oncology indicated that nurses believed that the quality of cancer care had decreased because of shorter hospital stays. These nurses, who worked in inpatient and outpatient settings, also indicated that both their workload and their paperwork had increased during the previous year. Table 6 compares the mean number of patients cared for and the perceptions of the appropriate mean number of patients cared for per shift per RN by employment setting and type of unit. In all cases, the mean number of patients cared for was greater than the number the RNs perceived as being appropriate. Most inpatient and outpatient RNs (72%) said they believed that double shifts and overtime work in response to staffing shortfalls harmed the care of cancer patients.^24^

**Table 6:** Mean Number of Patients Cared for Per Shift by Registered Nurses in the Inpatient or Outpatient Setting and by Oncology or Mixed Care Units^a^

Employment Setting	Mean No. of Patients Actually Cared for Per RN Per Shift	Mean No. of Patients Judged by RN as Appropriate Per Shift	Excess No. of Patients Per RN Per Shift
Inpatient dedicated oncology unit	5.26	3.97	1.29
Inpatient mixed unit	7.30	5.16	2.14
Outpatient dedicated oncology unit	18.70	12.25	6.45
Outpatient mixed unit	13.54	9.73	3.81

RN indicates registered nurse.

a Source: Lamkin 2002.^24^

#### Provider views on managed care impact on quality care

Four physicians, 3 nurses, and 3 business office staff members working with breast cancer patients were interviewed about their views on how managed care impacts cancer patients. Business office staff spent initial time helping the patients understand their insurance plans and how these plans affected their need for referrals and ancillary services. The nurses reported that a large portion of their time was spent calling insurance providers rather than providing patient care. The MCOs were viewed as very bureaucratic; nurses had difficulty contacting the right individual and getting answers to their questions. The purpose of many telephone calls was justifying why patients needed to have certain tests performed. Providers felt that care was fragmented and uncoordinated when a patient had to go to a primary care physician or to another physician's office for a referral, to another location for a test, and then back to the oncologist for radiation or chemotherapy. Providers also were concerned about their patients having to return to the primary care provider for follow-up care rather than being seen by the specialist. The providers thought that personnel at MCOs did not realize the burden that disjointed delivery of care placed on patients. Although most patients in the study were covered by managed care insurance, medical providers thought that the type of treatment received was based on medical necessity, and they did not believe that treatment was influenced by insurance type. In most cases, physicians did not know what type of insurance a woman had. However, physicians expressed concern about how the lowering of cancer drug reimbursements may affect drug choices in some practices.^18^ In a separate survey of oncologists, 58% said they considered revenue when making treatment recommendations.^25^ If a particular treatment would result in revenue loss, then most said they would choose to refer patients to a hospital (69%) or would prescribe an alternative medication (59%). In that survey, 76% of respondents worked in small community practices (1-3 physicians).

#### Pay for performance

A survey of 348 medical oncologists, radiation oncologists, and surgeons was conducted in California in 2000 to assess the use of financial incentives related to performance on quality measures. Overall, 35% of respondents reported being subject to incentives based on quality measures. Incentives were based on patient satisfaction surveys for 20% of the 348 respondents and on other quality measures, such as adherence to practice guidelines, for 15% of the respondents. Physicians most likely to be subject to quality incentives worked in staff-model or group-model health maintenance organizations or in large practices (>50 physicians). Quality incentives were more common among physicians who had a partial ownership interest in their practice, those who were paid predominantly by means other than fee for service, and those who were not reimbursed through capitation.^26^

### Managed Care/Reimbursement Perspective

Five sources presented views on quality cancer care from leaders in managed care industries (Table 7). The number of views represented was small; in 4 of the sources, only 6 leaders were interviewed or reported on their companies' strategies to address quality while controlling costs. The report of results from the survey commissioned by Genentech,^25^ which was mailed to nearly 4000 managed care professionals, did not specify the number of respondents.

**Table 7:** Sources Reporting Perceptions of Managed Care Professional About Quality Cancer Care

Reference Source	Study Design or Type of Publication	Type of Professional	Sample Size
Genentech 2008^25^	Survey/questionnaire	Medical directors, pharmacy directors, clinical pharmacists, other administrators; (>80% served on P&T committees)	NR^a^
NCCN 2009^27^	Interview	Senior managers from 3 managed care organizations	3
2008^30^	Interview	Industry thought leader (Director of Medical Oncology at Pittsburgh Medical Center Cancer Centers)	1
Wong 2008^28^	Recounting of innovation impact on quality of care	Vice president of pharmacy management at BCBS	1
Fenrick 2009^29^	Recounting of innovation impact on quality of care	Director of clinical pharmacy programs at BCBS of Florida	1
Total no. of managed care professionals/industry thought leaders			≥6^a^

P&T indicates pharmacy and therapeutics; NR, not reported; NCCN, National Comprehensive Cancer Network; BCBS, Blue Cross Blue Shield.

a The survey for Genentech was mailed to 3691 managed care professionals; the response rate was low, but the number of responses was not reported.

#### Cost-reduction strategies

Managing the availability of cancer treatment and individual patients' access to that treatment presents a significant challenge to payers as the costs of cancer interventions rise. Medical expenditures for cancer rank in the top 3 disease states for typical MCOs.^27^ Cancer chemotherapy represents a large share of drug costs to payers. For example, chemotherapy accounted for 35% to 40% of the $300 million in drug costs that were covered under medical benefits for 1 payer located in the mid-Atlantic region of the United States, and that payer's chemotherapy costs rose 25% each year.^28^ Payers are seeking cost-reduction strategies that still allow them to provide high-quality, optimal, evidence-based care. Payers are mindful of avoiding adversarial relationships with patient advocacy groups and the media^27^ and of making changes that lead to physicians withdrawing from their networks.^25^

Examples of specific strategies come from Blue Cross Blue Shield (BCBS) of Florida (BCBSF),^29^ which has developed frequently updated, detailed drug coverage guidelines; established its own care management program (rather than outsourcing this need); and set up an ambulatory infusion suite network that reportedly is more cost-effective than administering intravenous medications in a hospital. BCBSF also is developing retrospective drug use reviews to evaluate compliance with oral cancer drugs in the hope that better compliance will lead to better outcomes for patients with less cost to payers. To assist members, BCBSF is developing a self-service shopping tool that will allow members to view their copayment at various pharmacies before they fill an oral cancer drug prescription.

Clinical pathways are another tool that payers are using to control costs by reducing variation in care. The principle behind clinical pathways is that use of the same proven treatment regimens by all providers may produce more consistent outcomes and cost savings. Some of the money saved can be given back to providers as incentives for complying with the clinical pathways. The pathways are developed by clinicians without payer involvement, and compliance is monitored through electronic claims. A BCBS program (CareFirst) offers a higher reimbursement rate for chemotherapy agents if physicians have at least a 65% compliance rate with chemotherapy regimens for their patients overall. For supportive care, the minimum accepted compliance rate is 80%. BCBS does not plan to increase the minimum compliance for chemotherapy beyond 90% given the individualized nature of cancer treatment.^28^

A clinical pathway program at the University of Pittsburgh Medical Center uses a web-based decision support tool that is incorporated into the physician's workflow by containing the list of patients the physician will see and their histories related to the pathway. Decision aids include standard regimen-order sets, standard dose adjustments, patient education materials, and clinical trial literature related to the pathway regimens. This pathways program reportedly saved Highmark BCBS $1 million for a single biologic (bevacizumab; Genentech Inc., San Francisco, Calif) within 6 months.^30^

#### Increasing costs of oncology-related drugs

The NCCN discussed current practices with executives from 3 major MCOs to gain the payer's perspective on the rising cost of cancer treatment and to learn how patients, providers, and industry might be affected.^27^ Approaches varied among the 3 payers, with 1 payer imposing few restrictions on oncology-related drugs. That payer offered extensive coverage for cancer clinical trials and did not review or restrict access to experimental drugs when these were offered through its centers of excellence programs (practices that have high-volume experience with rare or complex cancers). The other payers required precertification, especially of expensive biologics. The second payer was considering proposals to manage cancer drug costs by establishing tiers for infusible drugs, which is a common practice with oral agents. In this approach, the patient's out-of-pocket costs increase with each tier level of the prescribed drug. This payer also was considering ways to encourage physicians to obtain drugs through a specialty pharmacy (eg, by limiting the reimbursement for a particular drug to what it would cost from the specialty pharmacy). The third payer more closely scrutinized the chemotherapy doses and the types of drugs used in combination regimens as a way to ensure that there was less variability in care. In keeping with reducing variability, this payer also was piloting an approach that reimbursed for an episode of care instead of directly reimbursing for infusion drugs.^27^

**Figure 2:**
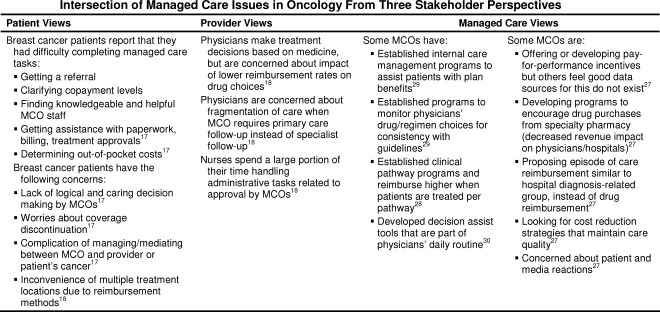
Managed care issues in oncology are listed from the point of view of patients, providers, and managed care organizations (MCOs).

In a survey conducted for Genentech, 62% of responding MCO professionals indicated that their companies had created or planned to create separate benefit designs for injectable specialty cancer drugs, putting them on fourth and fifth tiers (with higher cost sharing for patients).^25^ MCO professionals believed that prior authorizations were important or very important for limiting the use of biologics or injectables to indications approved by the US Food and Drug Administration (FDA) (79% of respondents) or to indications described in compendia (83%). Regarding reimbursements to practices for chemotherapy drugs, 53% of respondents had adopted or planned to adopt reimbursement based on average sales price, a method chosen by Medicare in 2005 that provides lower reimbursements than were paid historically. However, 57% of those who responded affirmatively to the question on average sales price did reimburse or planned to reimburse at higher rates than Medicare. Figure 2 provides a side-by-side summary of the patients', providers', and managed care professionals' perspectives on managed care in the oncology setting.

## DISCUSSION

In this literature review, we attempted to search the published literature comprehensively for stakeholder perceptions on quality of cancer care regarding chemotherapy; however, electronic searches have a limited capacity to capture all citations on a topic.^31^ Despite this limitation, the small number of studies that we identified probably was because of the paucity of scientific exploration on the topic. High-quality cancer care cannot be achieved without an understanding of perceptions of quality. Efforts to understand stakeholder needs better are even more essential during this time of change in the US healthcare system to assure that quality of cancer care is maintained and improved.

The quality of cancer care is under pressure in part because of the rising number of cancer patients in the United States.^32^ Efforts to deliver quality cancer care also will be challenged increasingly by a shortage of practitioners and support staff,^33^ especially as the administrative burden of managed care increases. By 2020, it is expected that the shortage of oncologists in the United States will be between 2350 and 3800, which represents a capacity of between 9.5 million and 15 million office visits. This workforce shortage largely will be caused by a slower increase in the number of oncologists compared with the increased demand.^4^,^5^

To compensate for physician shortages, nurse practitioners and physician assistants likely will be used increasingly in the future.^34^ Education programs must be developed to address cancer-specific procedures, clinical situations, and reimbursement issues as the roles of these professionals in cancer care increase.^35^,^36^

The personal interactions that patients desire will require changes in managed care processes and systems for information gathering that increase the availability of providers to care for patients. The assignment of case managers can help patients navigate the extensive number of clinical, financial, and reimbursement decisions; however, this requires sufficient training of personnel, more uniformity in procedures, and adequate funding by insurers. Scientific evidence that case management can increase the efficiency and quality of cancer care continues to be important in bringing about change in the healthcare system.

Current national legislative efforts to reform the US healthcare system have several different goals, among which are to increase access to affordable health insurance and to control ongoing increases in healthcare costs. The development and marketing of new cancer therapies does not promise to make controlling healthcare costs any easier. The cost of innovative treatments is a concern for payers, although clinical evidence often indicates that the use of these treatments is appropriate. Payers are concerned that physicians may choose the regimen that provides greater revenue for their practice, whereas physicians have expressed (see Provider Prospective, above) that their concern is with revenue loss from certain therapies, which can make it difficult to sustain their practices.^25^,^37^

Current cancer drug reimbursement is rooted in the system of compensating physicians for their time, which relies on the Resource-Based Relative Value Scale (RBRVS). This system discriminates against services in which cognition is dominant, favoring reimbursement of procedures and imaging over tasks such as supervising nurses, contacting other treating physicians, and checking laboratory results.^38^ In the past, when oncologists could make up lost earnings through higher drug reimbursements, they were not as concerned about the issues with the RBRVS system. After Medicare tied drug reimbursements to the actual average sales price of drugs in 2005, profit margins on chemotherapy decreased, and oncologists could no longer compensate for lost earnings.^38^ Nongovernment payers were slow to adopt Medicare reimbursement methods, but a growing number of those payers planned to implement these changes in 2007 and 2008.^39^

Innovative cancer drugs can cost payers an additional $10,000 to $20,000 or more for just the initial phase of treatment,^40^ with higher costs incurred if the agent is administered chronically. Payers require prior authorizations to ensure that these drugs are used according to FDA guidelines or compendia recommendations. However, the cost of chemotherapy or supportive drugs increases indirectly when considerable time is required to fill out extensive prior authorization forms.^37^ In addition, requiring prior authorizations for various treatments reduces the time that providers, especially nurses, can spend with patients, which may negatively affect providers' perceptions of the quality of care they administer.^18^

Patients' abilities to comply with oncology prescriptions also are affected by insurance policies, such as high coinsurance. If patients cannot afford to pay their part, then they may have to take a different medication that may not be as effective, or they may not receive an oncologic agent to treat their disease.

Physicians and patients may value newer agents and regimens that extend life longer than established therapy but that still do not offer substantial gains in life expectancy (eg, just a few months of extra survival time). Patients with cancer may be willing to take substantial financial risks to extend their lives.^32^ However, many patients have fewer financial resources to risk; and, from an ethical standpoint, access to beneficial therapies should not be restricted to wealthier patients.^41^ Instituting measures to satisfy the perceived needs identified in this study are a way to assure improved quality of cancer care.

## CONFLICT OF INTEREST DISCLOSURES

Eli Lilly and Company provided funding in support of this research. Drs. Pohl, Liu, and Peltz are employed by Eli Lilly and Company and own stock in the company. Dr. Colosia, Ms. Copley-Merriman, Ms. Khan, and Dr. Kaye are employed by RTI Health Solutions, a business unit of RTI International.
